# The case against probabilistic inference: a new deterministic theory of 3D visual processing

**DOI:** 10.1098/rstb.2021.0458

**Published:** 2023-01-30

**Authors:** Fulvio Domini

**Affiliations:** CLPS, Brown University, 190 Thayer Street Providence, Rhode Island 02912-9067, USA

**Keywords:** depth perception, cue integration, 3D vision, computational models

## Abstract

How the brain derives 3D information from inherently ambiguous visual input remains the fundamental question of human vision. The past two decades of research have addressed this question as a problem of probabilistic inference, the dominant model being maximum-likelihood estimation (MLE). This model assumes that independent depth-cue modules derive noisy but statistically accurate estimates of 3D scene parameters that are combined through a weighted average. Cue weights are adjusted based on the system representation of each module's output variability. Here I demonstrate that the MLE model fails to account for important psychophysical findings and, importantly, misinterprets the just noticeable difference, a hallmark measure of stimulus discriminability, to be an estimate of perceptual uncertainty. I propose a new theory, termed Intrinsic Constraint, which postulates that the visual system does not derive the most probable interpretation of the visual input, but rather, the most stable interpretation amid variations in viewing conditions. This goal is achieved with the Vector Sum model, which represents individual cue estimates as components of a multi-dimensional vector whose norm determines the combined output. This model accounts for the psychophysical findings cited in support of MLE, while predicting existing and new findings that contradict the MLE model.

This article is part of a discussion meeting issue ‘New approaches to 3D vision’.

## 1. Introduction

**Figure 1.**
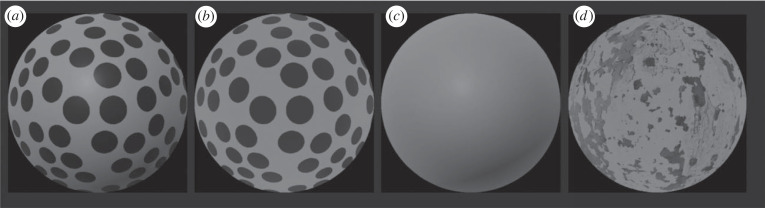
Renderings of the same 3D surface with different 3D information: texture and shading (*a*), only texture (*b*), only shading (*c*) and ineffective texture (*d*).

The spatial organization of the visual world in three dimensions is a fundamental aspect of our conscious perception. The objects we interact with appear to have a definite and unambiguous 3D structure. This appearance, however, hides the inherent ambiguity of the retinal stimulation, since there are in principle infinite 3D configurations that may have produced the same retinal image and infinite images that may arise from the same 3D configuration. Take for instance the picture on figure 1*a*: it is immediately perceived as a smooth bump ‘bulging out’ of the image plane. What we see, however, is only one of infinite structures that may have produced that projection. Selecting only one of these possible interpretations is therefore a central concern of any theory of 3D vision.

A prominent theory that has dominated the field of vision science for over two decades addresses this issue as a problem of probabilistic inference. This theory postulates that among all possible interpretations of an image the visual system chooses that most probable [1–37]. This theory further assumes that the visual system is endowed with specialized and independent visual modules that choose the most probable depth interpretation from individual *depth cues*, for example, the texture and shading patterns on figure 1*a*. Since these interpretations are only ‘best guesses’ of what is ‘out there’, the outputs of the depth modules randomly and independently fluctuate across views of the same distal structure. These fluctuations are often referred to as estimation noise. Nevertheless, an important assumption of the theory is that, despite estimation noise, the outputs from these modules (i.e. their 3D estimates) are *unbiased*. This means that the *average* output of the modules (or, more generally, the expected value) coincides with the ground truth. Probabilistic theories (e.g. Bayesian theories) of *cue integration* specify how unbiased outputs should be combined in a statistically optimal manner, which results in the reduction of the estimation noise affecting each individual output. Specifically, the maximum-likelihood estimation (MLE) model combines the unbiased outputs of individual modules through a *weighted* average, where larger weights are assigned to less noisy outputs. In addition to the assumption of unbiased outputs, this model therefore requires that depth modules provide explicit information about their output variability [15] (e.g. by encoding the noise distribution with neural populations).

These fundamental assumptions, however, are problematic. First, the assumption that outputs are unbiased has been falsified in numerous studies [38–66]. An informal test can be done by directly observing figure 1. Here the four pictures are renderings of the same underlying 3D structure and yet they are all perceived to have a different 3D structure. For example, the images containing only shading or texture information look flatter than the image containing both shading and texture information. Moreover, the surface with polka-dot texture (figure 1*b*) looks deeper than the one with only shading information (figure 1*c*) and also deeper than the surface covered with a less effective texture pattern (figure 1*d*). Second, the assumption that module outputs are noisy also clashes with simple phenomenological observations. Repeated viewing of any of the three surfaces in figure 1 does not seem to produce any detectable changes in their perceived 3D structure. This is also echoed in the fact that the perceived shape of objects in the real world appears stable and unambiguous and does not appear to change over successive viewings. In this paper, I will describe a new theory of cue integration that does not require any of the assumptions of the probabilistic inference model of depth cues and depth-cue integration. First, the new theory assumes that the output of individual depth modules is *linearly* related to distal 3D properties, but that this linear relationship is not necessarily accurate. Second, it assumes that this linear mapping between distal 3D properties and module outputs is *deterministic*, since the ‘noise’ intrinsic to this mapping is negligible and independent of the particular visual stimulus. The two theories also disagree fundamentally in postulating the source of module output variability. Probabilistic theories assume that visual modules reverse the process of image formation by finding all the 3D structures in the world that may have produced the retinal input and picking among those the most probable. If a cue is unreliable then even small variations of the visual input results in large variations of the output. Specifically, an ‘unreliable’ cue is one which provides only a weak signal regarding the 3D property, therefore eliciting a ‘noisy’ or more variable response. The model proposed here, in contrast, assumes linear input–output mappings that are stable, but often inaccurate, where in most cases individual modules result in an underestimate of distal properties. According to the new theory ‘noise’ or variability is not related to the space of possible 3D interpretations, where ‘weak’ cues accommodate a larger range of possible 3D interpretations, and are therefore deemed ‘noisy’. Instead, a weaker cue does not generate a noisier output, but generates an output with a shallower linear relationship between distal 3D properties and the module output. This is because the image measurement that carries 3D information from that cue delivers a small readout. For instance, 3D information from texture is given by the *texture gradient*, which quantifies the rate of change of the two-dimensional shape of texture elements on the image. In figure 1*b*, the texture gradient is larger than the texture gradient in figure 1*d*, hence the texture cue in figure 1*b* is *stronger* than the texture cue in figure 1*d*. Note, however, that figure 1*d* does not look ‘noisier’ than figure 1*b*, but it definitely looks shallower. For the new theory the source of a module output variability is the change of ‘nuisance’ parameters such as the object material properties, the illumination conditions, the object distance from the observer, etc., which can in principle cause sizable changes in the cue strength. In the examples of figure 1, the nuisance variable affecting the texture cue is the material composition of the objects and the nuisance variable affecting the shading cue is the illumination condition. The new theory, name Intrinsic Constraint (IC) theory, postulates that processing of independent depth modules and their combination thereof, is done in a way to minimize the sensitivity to nuisance parameters while maximizing the response to changes of distal 3D properties. The combination of module outputs is achieved by the *Vector Sum* model, which combines the output of individual modules through a vector sum. In contrast with the MLE model, this vector sum does not weigh the individual module outputs and, therefore, does not require any estimate of variability or strength of individual cues. The Vector Sum model predicts systematic biases arising from cue combination since combining cues results in a stronger combined signal, and, therefore a larger magnitude in the estimate. As a consequence, combined-cue stimuli always appear deeper than single-cue stimuli, a phenomenon that can be readily observed on figure 1 by comparing the texture-shading stimulus (figure 1*a*) with the texture-only (figure 1*b*) and shading-only (figure 1*c*) stimuli.

In addition to not requiring an internal estimate of cue variability, the IC theory and the Vector Sum model have also a theoretical advantage over probabilistic theories and the MLE model. Probabilistic theories need to clarify how the visual system has learnt either through evolution or during development to associate the visual input to the accurate geometrical description of the viewed objects. In fact, the brain has no way to access the geometrical structure of the outside world since all information comes through our inherently ambiguous proximal sense data. The IC theory, instead, is based on the conjecture that the visual system ‘discovers’ (ontogenetically of phylogenetically) distal properties of objects from the *intrinsic* relationships existing among independent image cues. In principle, the correlation among these cues enables the bootstrapping of individual depth modules so as to produce outputs that are *linearly* related to distal properties. This learning process is therefore unsupervised, since it does not require explicit access to the ground truth. Although the existence of this unsupervised learning process is a conjecture that needs to be verified through machine learning algorithms, there is already evidence that unsupervised learning can be very effective in spontaneously clustering images according to distal properties such as reflectance and illumination [67–69]. The Vector Sum model is also more parsimonious than the MLE model since (i) it assumes a linear input–output mapping instead of an accurate or veridical mapping, which can account for the biases observed in depth estimation, and (ii) it does not require an estimate of the output variability of individual modules.

Finally, there is a major difference in methodological assumptions at the heart of the two theories. According to the probabilistic inference theory and MLE model, variability in perceptual estimates in psychophysical tasks as measured by the just noticeable difference (JND) directly reflects the internal ‘noise’ or reliability of module outputs or cues. Instead, according to the IC theory and Vector Summation model (which assume that outputs of depth modules are not stochastic but deterministic), variability in perceptual estimates is simply a reflection of task-specific demands, such as memory limitations intrinsic to psychophysical tasks. Instead, differences in measured JNDs depend simply on the differences in strength of the linear mapping between distal property and of single or combined module outputs. Shallower mappings result in higher JNDs while steeper linear mappings result in lower JNDs. Thus, JNDs do not appear in the main equation of the Vector Summation model.

In the following sections, I will detail the differences between the IC theory and Probabilistic Inference theory. In §2, I first describe the differences between Vector Sum and the MLE models. In §3, I will describe an entirely new interpretation of JND compatible with the Vector Sum assumption that the output of depth modules is not based on a stochastic process that depends on the stimulus reliability, as assumed by the MLE. I will then describe a novel methodology to test the validity of this new interpretation of JND and empirical evidence that supports it. In §4, I will show new results indicating that the output of single-cue modules is systematically biased and provide further empirical evidence that corroborates the predictions of the Vector Sum model. Finally, in §5, I will describe results of a classic cue integration experiment that quantitatively agree with the predictions of the Vector Sum model and, at the same time, falsify the predictions of the MLE model.

## 2. Maximum-likelihood estimation versus Vector Sum model of depth-cue integration

**Figure 2.**
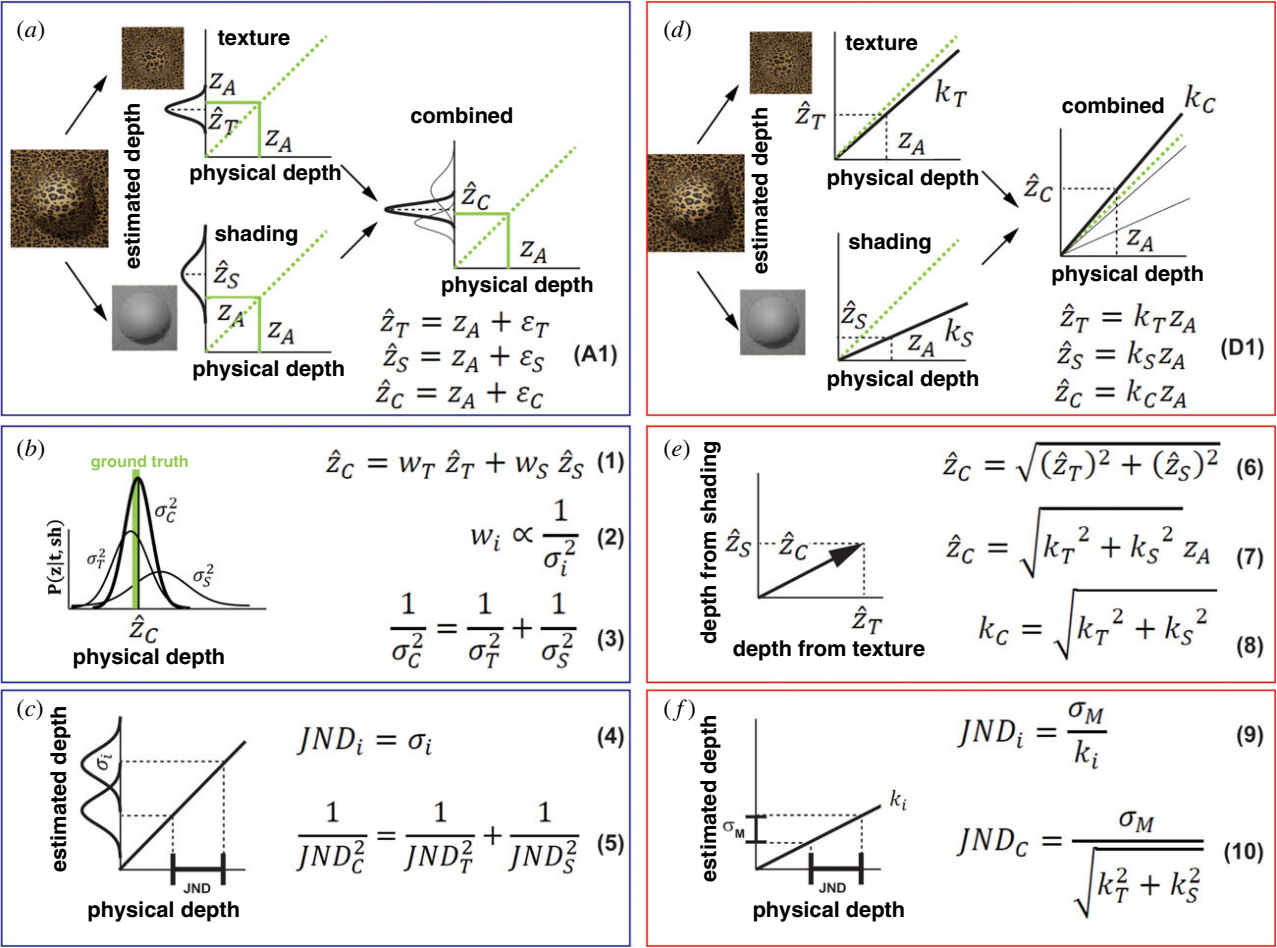
(*a*) The MLE model maps 3D properties (horizontal axes) to estimated properties (vertical axes) with probability distributions (solid black). Individual distributions are associated with independent cues in an image (left), which in this example are texture (top) and shading (bottom). The probability distribution of the combined estimate is sharper than those of individual estimates (right). Although the distributions associated with a particular visual input are peaked at values different from the ground truth *z_A_*, they are on average accurate. (*b*) For linear MLE models, the combined likelihood function is the product of the individual likelihood functions (left) and can be obtained through a weighted average of single-cue estimates, where the weights are inversely proportional to the standard deviation of the noise affecting each cue (equations (1) and (2)). The result is a less noisy combined estimate (equation (3)). (*c*) For MLE models, the JND obtained in discrimination tasks is a proxy measure of the standard deviation of the likelihood function (left, equation (4)). JNDs can therefore be used to predict the s.d. of the combined estimate (equation (5)). (*d*) The Vector Sum model maps distal 3D properties to 3D estimates with a deterministic mapping. Assuming a linear function, the slope of this function is not unitary, as for MLE models, but depends on the strength of individual cues. In this example, texture (top left) is stronger than shading (bottom left). The strength of the combined-cue (right) is always larger than that of the single-cues. (*e*) For the Vector Sum model, individual cue estimates are the components of a multi-dimensional vector (left). The combined estimate is therefore the vector sum of the individual estimates (equations (6) and (7)) and its strength (slope) is also the vector sum of the individual strengths (equation (8)). (*f*) For the Vector Sum model, the noise in a discrimination task is related to memory encoding and retrieval and it is independent of the particular stimulus. The JND measures the change in the distal depth producing a change in perceived depth sufficient to overcome this noise (left). The JND is therefore inversely related to the strength of the stimulus (equation (9)). This explains why the JND for combined stimuli is smaller than the JND of single-cue stimuli, since the strength of combined stimuli is always larger (equation (10)). This prediction is formally identical to that of the linear MLE models (equation (5)).

Probabilistic models provide mathematical methods for assigning a probability value to each 3D interpretation of an image based on knowledge about the image formation process and prior statistical knowledge of the material composition of objects in the world, their structure and the viewing parameters [15]. As depicted in figure 2*a* (top left), this probabilistic mapping between the distal depth values (horizontal axis) and possible perceptual interpretations (vertical axis) is represented as a probability distribution (solid black). If we disregard the influence of prior knowledge, this probability distribution coincides with the *likelihood* function. MLE models assume that the *most likely* 3D structure determines our perception [15]. In figure 2*a* (top), this perceptual solution is the peak of the probability distribution. Note that this choice is only a ‘best guess’, since it often does not correspond to the ground truth (*Z_A_*, indicated in green). However, if the same depth-from-texture analysis is repeated over a large number of images of the same 3D surface then the average estimate will correspond to the ground truth. This is a fundamental assumption of MLE models: *3D estimates are unbiased* [15]. The extent to which these solutions fluctuate around the ground truth depends on the *reliability* of the texture pattern, determined by how sharply peaked the probability distribution is. Another image pattern carrying 3D information that can be seen on the rendering of figure 2*a* is the *shading gradient*, determined by the smooth luminance change across the image (figure 2*a*, bottom left). In the example, the shading gradient is characterized by a wider probability distribution. In summary, the module outputs are modelled as unbiased estimates with additive noise reflecting their *reliability* (figure 2*a*, equations (A1)). The MLE model combines the individual outputs in a statistical optimal fashion in order to minimize the combined output noise [15]. In the example of figure 2*a*, combining the texture and shading cues produces a sharper probability distribution whose peak is on average closer to the ground truth. Since the two cues are independent, the combined probability distribution is simply the product of the two individual probability distributions (figure 2*a*, right). This is achieved through a weighted average where the weights are inversely proportional to the variance of the noise of the single-cue estimates (figure 2*b*, equations (1) and (2)). The variance of the combined estimate is smaller and can be predicted from the variance of the single-cue estimates [3] (figure 2*b*, equation (3)).

**Figure 3.**
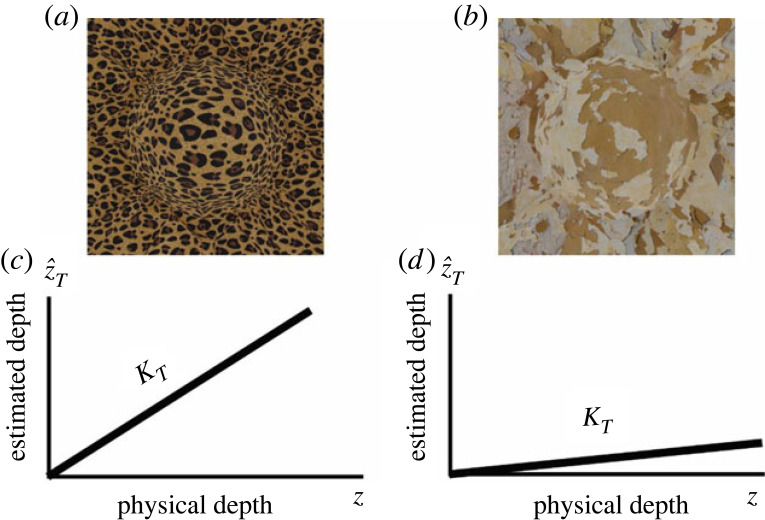
(*a*,*b*) 3D information from individual cues varies across images. (*c*,*d*) Assuming a linear relationship between a 3D estimate $({\hat{z}}_{T})\;$ and the encoded distal property (*z*), a degraded input results in a smaller slope (*k_T_*) of this relationship. Termed strength, this slope is larger for the strong texture pattern on the left than for the weak texture pattern on the right.

**Figure 4.**
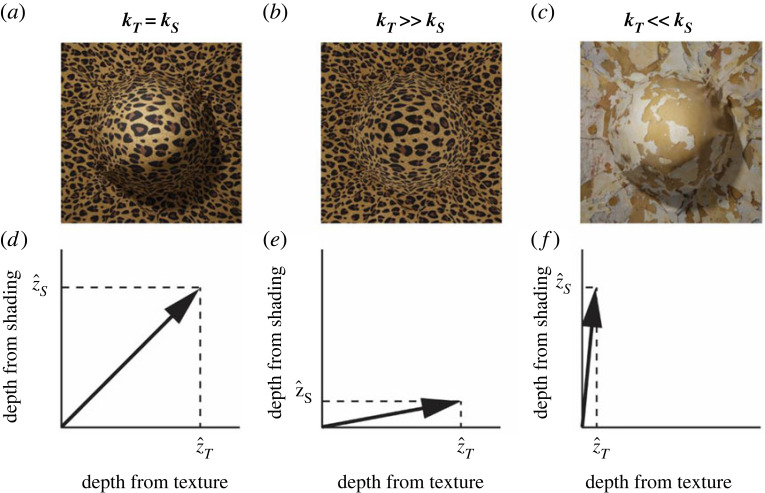
(*a–c*) The same 3D structure as in figure 3 rendered with different combinations of texture and shading information. (*d–f*) The vector sum of individual cue estimates changes among the three viewing conditions, but this change is smaller than the relative change of each single-cue estimate.

The Vector Sum model, in contrast, assumes a *linear* mapping between distal 3D properties and the output of individual modules (figure 2*d*, left). This assumption is based on the conjecture that the developing visual system may learn statistical co-occurrences among independent image patterns and attribute *these co-occurrences* to external causes encoded as latent variables (e.g. a depth map). Since this learning process does not correlate image cues with the ground truth, the linear mapping is in general not accurate (i.e. the slope is not 1) and, instead, depends on the ‘quality’ of the visual input. Consider for instance in figure 3, the two images of the same 3D bump arising from objects of different material compositions. Whereas the image on the left is immediately perceived as a protruding surface, the image on the right appears almost flat because it displays a much weaker texture gradient. This degraded input will yield a shallow linear function. I term the slope of this function *cue strength*. In the example of figure 3, the *strength* of the texture cue is therefore the slope *k_T_* of the observed mapping between the ground truth *z* and the texture output ${\hat{z}}_{T}$. Therefore, *k_T_* is much smaller for the image on the right of figure 3 than for the image on the left. What is evident from this example is that the magnitude of the output of single-cue modules depends both on distal depth and the cue strength. In turn, cue strengths depend on nuisance scene parameters such as the material composition of objects, ambient illumination, observer's motion, etc. A desirable cue combination rule is therefore one that maximizes the sensitivity to distal 3D properties while at the same time minimizing the influence of nuisance scene parameters. This can be achieved by considering the individual depth estimates as scalar components of a multi-dimensional vector, where the length of the vector corresponds to the combined-cue depth (and the orientation is ignored). Consider figure 4 for the particular case of texture and shading information. For illustration purposes suppose that in the stimulus on the left, texture and shading have the same strength. This means that the output of the texture module (${\hat{z}}_{T}$) and the output of the shading module (${\hat{z}}_{S}$) are the same (figure 4*a*). In the stimulus in the centre, the strength of texture is much larger than the strength of shading. In the stimulus on the right, the strength of shading is much larger than the strength of texture. It can be shown mathematically that if the axes of this multi-dimensional space of cue estimates are scaled to take into account the natural variability of the scene parameters associated with each individual cue, then the length of the multi-dimensional vector will be maximally sensitive to changes in distal depth values and minimally sensitive to variations of the strength of image cues (see Appendix A for the mathematical proof).

To recap, the Vector Sum model represents single-cue estimates ${\hat{z}}_{i}=k_{i}z$ as the components of a multi-dimensional vector (figure 2*e*). The magnitude of this vector ${\hat{z}}_{C}$ is the combined estimate described by the *Vector Sum equation*:

$${\hat{z}}_{C}=\sqrt{{\left({\hat{z}}_{1}\right)}^{2}+{\left({\hat{z}}_{2}\right)}^{2}+\ldots {\left({\hat{z}}_{n}\right)}^{2}}=\sqrt{{\left(k_{1}z\right)}^{2}+{\left(k_{2}z\right)}^{2}+\ldots {\left(k_{n}z\right)}^{2}}.$$

In summary, the MLE model and Vector Sum models describe different mechanisms of 3D processing based on fundamentally different assumptions about how the visual system deals with the ambiguity of the visual input.

The MLE model assumes that the goal of the visual system is to provide a 3D interpretation of the visual input that is as close as possible to the ground truth. Independent modules output the most likely interpretation from single cues. Repeated viewing of a series of equally reliable stimuli will cause the output to ‘jump around’ the ground truth. Since on average the output coincides with the ground truth (i.e. it is unbiased), the MLE model combines cues in order to minimize the variability of the combined output.

The Vector Sum model strives for the most stable interpretation of the visual input amid variations of viewing conditions. Single cues deliver a 3D output that is linearly related to the distal property. This linear function depends on the *cue strength*. For instance, a cue that is referred to as ‘unreliable’ or ‘noisy’ in the MLE model is instead, according to the Vector Summation Model, one that yields a shallow slope of the linear function. In contrast with the MLE model, the input–output mapping is *deterministic*, only affected by negligible noise that is stimulus-independent Therefore, repeated viewing of stimuli with the same strength will yield a nearly constant output (negligible noise). The output only changes when the stimulus itself changes, for example for a given distal 3D structure in response to changes in viewing-dependent nuisance parameters (viewpoint, lighting, material pattern, etc.). The goal of the Vector Sum rule of cue combination is to minimize the influence of the nuisance parameters on the 3D derivation process.

In the following sections, I will describe in detail how these different assumptions about the mechanisms of 3D processing can be tested and provide converging empirical evidence confirming the predictions of the Vector Sum model.

## 3. What is the source of perceptual variability according to maximum-likelihood estimation and Vector Sum models? Interpretation of just noticeable differences

### (a) Maximum-likelihood estimation model and Vector Sum model interpretation of just noticeable difference

The most important empirical tests of the MLE model have used discrimination thresholds to measure the variability of depth judgements. These thresholds, termed JNDs, are usually measured with a 2 Alternative Forced Choice (2AFC) task where a standard stimulus, which is kept fixed throughout the experiment, is being compared to a comparison stimulus that the experimenter varies either through a staircase procedure or through a range of constant stimuli. The discrimination threshold is the minimum difference in depth magnitude between the comparison and standard yielding a reliable discrimination (e.g. 84% correct discriminations). After I summarize the interpretation of JND according to the MLE model, I will describe an alternative interpretation compatible with the assumptions of the Vector Sum model.

**Figure 5.**
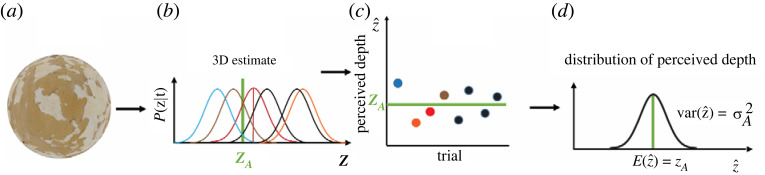
When the same unreliable texture stimulus (*a*) is viewed on different trials, it will determine likelihood functions peaked at different depth values. In this example, these functions, depicted in different colours, are assumed to be Gaussian (*b*). If perceived depth is predicted to be a MLE then it will vary from trial to trial (*c*). The distribution of depth estimates is in this case also Gaussian, centred at the veridical depth value *z_A_* and with variance $\sigma_{A}^{2}$equal to the variance of the likelihood functions (*d*).

The MLE model derives from the input the most likely 3D interpretation that varies across repeated views of similar inputs to an extent that depends on the stimuli reliability. An important prediction of this model is, therefore, that the variability of the MLE interpretation causes the variability of depth judgements. To illustrate, consider the plaster texture in figure 5*a*. The likelihood function associated with this pattern is expected to be wide since there are many possible 3D surfaces that are likely to have produced that pattern [13,23]. Due to this ambiguity of the plaster texture, slightly different texture patterns will yield likelihood functions that have different peaks (figure 5*b*). Therefore, the MLE output of modules will vary considerably across trials and, consequently, also the predicted perceptual judgements (figure 5*c*). If the likelihood distribution is Gaussian, then the distribution of depth judgements will also be Gaussian with the same variance and centred at the true depth value (figure 5*d*). If the same experiment is repeated with the cheetah-texture pattern (figure 3*a*), then the MLE model predicts a much smaller variation in depth judgements, since the cheetah texture is more reliable and therefore characterized by a much sharper likelihood. Since the distribution of depth judgements is assumed to be unbiased, in a discrimination task, the JND will coincide with the discriminable separation between the means of the two distributions being compared (figure 2*c* left, equation (4)).

By contrast, for the Vector Sum model, the input–output mapping of visual modules is *deterministic*. Consequently, an observer is expected to perceive the same depth magnitude from a sequence of equivalent stimuli (e.g. repeated viewing of the plaster stimulus). Most importantly, whatever negligible amount of neural noise may affect the output, it is independent of the 3D information contained in the input. In other words, a sequence of cheetah-texture stimuli will produce the same output variability as a sequence of plaster-texture stimuli. This basic assumption of the Vector Sum model requires a radical re-interpretation of JNDs from that of the MLE model. The Vector Sum model assumes that the source of noise that determines the JND is entirely task-related. For instance, in a 2AFC task, the observer must store in memory the perceived depth magnitude of the stimulus in the first interval and then retrieve this information to compare it with the perceived depth magnitude of the second stimulus. We can assume that this process of memory storage and retrieval introduces noise to the *perceived* depth estimate of the first stimulus, a sort of memory ‘smearing’ with s.d. $\sigma_{M}$. Therefore, the difference of perceived depth between the two stimuli $(\Delta \hat{z})\;$ must be large enough to overcome this task-related memory noise $\left(\Delta \hat{z}=\sigma_{M}\right)$. As just mentioned above, what is fundamental here is that according to the Vector Sum model this difference is *independent* of the type of stimuli being discriminated. For instance, a cheetah texture and a plaster texture must bring about the same perceived depth difference $\Delta \hat{z}$ for a reliable discrimination. However, since the JND is defined in terms of *distal* depth values, the change in simulated depth of the cheetah-texture stimulus $(\;\mathrm{JN}D_{\mathrm{cheetah}})$ producing a perceived depth difference $\Delta \hat{z}=\sigma_{M}$ depends on its strength ($k_{\mathrm{cheetah}}$): $\Delta \hat{z}=k_{\mathrm{cheetah}}{\mathrm{JND}}_{\mathrm{cheetah}}$. In the same way, $\Delta \hat{z}=k_{\mathrm{plaster}}{\mathrm{JND}}_{\mathrm{plaster}}$. Therefore, the two stimuli yield the same perceived depth difference $\Delta \hat{z}$ if ${\mathrm{JND}}_{\mathrm{cheetah}}<{\mathrm{JND}}_{\mathrm{plaster}}$ since $k_{\mathrm{cheetah}}>k_{\mathrm{plaster}}$. An important consequence of this interpretation of JND is that it is inversely proportional to the strength of a stimulus (figure 2*f*, equation (9)).

In summary, the interpretations of JND according to the MLE and Vector Sum models are profoundly different since they assume different sources of noise affecting the JND. Nevertheless, in specific experimental conditions they make the same predictions. For instance, both models predict that discriminating cheetah-texture stimuli yields a smaller JND than discriminating plaster-texture stimuli. This is because, on the one hand, the MLE model assumes that cheetah-texture stimuli produce a less variable output than plaster-texture stimuli whereas, on the other hand, the Vector Sum model assumes that the strength of cheetah-texture stimuli is larger than the strength of plaster-texture stimuli. In the next sections, I will describe a novel methodology that allows us to discriminate between the two models.

### (b) Just noticeable difference according to the maximum-likelihood estimation and Vector Sum models: empirical evidence with single cues

**Figure 6.**
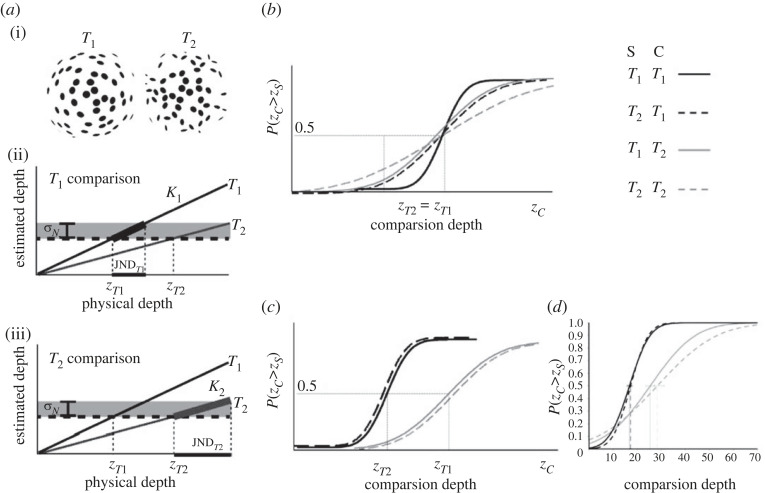
(*a*) Two texture stimuli of different strengths (*T*_1_ and *T*_2_, (i)). For the Vector Sum model, the JND measured in a discrimination task only depends on the strength of the comparison stimulus. If the comparison is the strong stimulus (*T*_1_, (ii)) then the JND, equal to the distal depth difference necessary to overcome the task-related noise ($\sigma_{N}$), will be smaller than the JND obtained when the comparison stimulus is the weak stimulus (*T*_2_, (iii)). (*b*) Psychometric functions predicted by the Bayesian model (the curves are for illustrative purposes only and not based on actual data). In the legend S indicates the standard stimulus and C the comparison stimulus. The slope of the psychometric functions depends on both the comparison and standard stimuli. (*c*) Psychometric functions predicted by the Vector Sum model. The slope of the psychometric functions only depends on the comparison stimulus. (*d*) Empirical data [70].

Fundamental to the following discussion is how the two models interpret the role played by the standard stimulus, which is kept fixed during a discrimination task, and the comparison stimulus, which is varied through a staircase procedure or the constant stimuli method. I will now show that the two models predict the same JNDs only in experimental conditions where the standard and comparison stimuli contain the same cue or set of cues (e.g. discriminating two cheetah-texture stimuli). When 3D information composing the standard and comparison differs (e.g. discriminating a cheetah-texture stimulus from a plaster-texture stimulus) the predictions of the two models diverge. Figure 6*a*(i) shows a stimulus *T*_1_ that is more informative than a stimulus *T*_2_. In what follows I will describe the predictions of the two models for a discrimination task in all possible conditions where each of the stimuli *T*_1_ and *T*_2_ are either assigned to be the fixed standard or the variable comparison. In particular, I will formulate the specific predictions for the point of subjective equality (PSE) and *slope* of the *psychometric* function, which relates the depth of the comparison stimulus to the proportion of responses where the comparison is perceived to be deeper than the standard.

Figure 6*b* shows the predictions of the MLE model for all possible combinations of standard and comparison stimuli. The first prediction is that all the psychometric curves have identical PSEs since the MLE model predicts that the perceived depth of the two texture stimuli is the same when the distal depth is the same (figure 6*b*, PSEs: $z_{T1}=z_{T2}$). Second, the slope of the psychometric function should depend on the reliability of *both* the fixed standard and the varying comparison. This is because the depth estimates of both the standard and comparison stimuli are subject to estimation noise. As discussed in §3(a), these are described by probability distributions centred at the simulated depth values with s.d. reflecting the stimuli reliabilities. In the example of figure 6, the texture stimulus *T*_1_ is more reliable (i.e. less noisy) than the texture stimulus *T*_2_. Judging above chance which stimulus is deeper requires a depth difference between the simulated depth of the two stimuli that separates the means of these probability distributions by an amount overcoming the pooled variance (i.e. the sum of squares of the two variances). For instance, if a reliable comparison is being discriminated from a reliable standard then this difference will be smaller than when it is compared to an unreliable standard. Therefore, in the first case, the slope of the resulting psychometric function will be larger than in the second case. In summary, according to the MLE model, we should expect (i) the steepest slope when standard and comparison have both high reliability (figure 6*d*, solid black); (ii) intermediate slopes when standard and comparison have different reliabilities (figure 6*d*, dashed black and solid grey); and (iii) the shallowest slope when both standard and comparison are unreliable (figure 6*d**,* dashed grey).

For the Vector Sum model, the strength of the texture stimulus *T*_1_ is larger than the strength of the texture stimulus *T*_2_. According to the Vector Sum model (i) 3D estimates from both standard and comparison stimuli are deterministic, that is, they do not vary across trials, and (ii) depth discrimination is achieved when the perceived depth of the comparison stimulus differs from the perceived depth of the standard stimulus by an amount that overcomes the task-related noise $\sigma_{M}$. The Vector Sum model therefore predicts that the comparison stimulus is what determines the JND. Consider figure 6*a*(ii) where *T*_1_ is the comparison stimulus and *T*_2_ is the fixed standard. First, note that a perceptual match (horizontal dashed line) is achieved when the simulated depth values of the two stimuli are different ($z_{T1}$ and $\;z_{T2}$ in the figure), because they have different cue strengths. Second, for a measurable discrimination, the simulated depth of *T*_1_ (comparison) must increase from the pedestal value $z_{T1}$ by an amount yielding a perceived depth difference equal to the standard deviation of the task-related noise $\;\sigma_{M}$. This amount is the JND (${\mathrm{JND}}_{T_{1}}$, figure 6*a*(ii), black horizontal bar on the *x*-axis), which we saw is inversely proportional to the cue strength (figure 1*f*, equation (9)). If the comparison is the weaker stimulus (i.e. texture *T*_2_) then the *JND* will be larger (${\mathrm{JND}}_{T_{2}}$ on figure 6*a*(iii)). In figure 4*c*, I show the predicted psychometric functions based on the IC model hypothesis that the JND only depends on the cue strength *k* of the comparison stimulus. When the comparison is the strong-cue stimulus, we expect *steep* and identical psychometric functions when both (a) the standard is a strong-cue stimulus (solid black) and (b) the standard is a weak-cue stimulus (dashed black). Similarly, when the comparison is the weak-cue stimulus, we expect shallow and identical psychometric functions for both a strong (solid grey) and a weak (dashed grey) standard. The results of a recent study shown in figure 6*d* confirm the predictions of the Vector Sum model and clearly disagree with those of the MLE model [70].

### (c) Just noticeable difference according to the maximum-likelihood estimation and Vector Sum models: empirical evidence with multiple cues

The MLE model and Vector Sum model predict different outcomes of cue integration with regard to the variance of the combined output. The MLE model predicts that combining cues yields a reduction of the variance of the combined output (figure 2*b*, equation (3)) [3]. This prediction has been tested extensively in depth-discrimination experiments where standards and comparisons are the same stimulus type: either single-cue stimuli or combined-cue stimuli.

**Figure 7.**
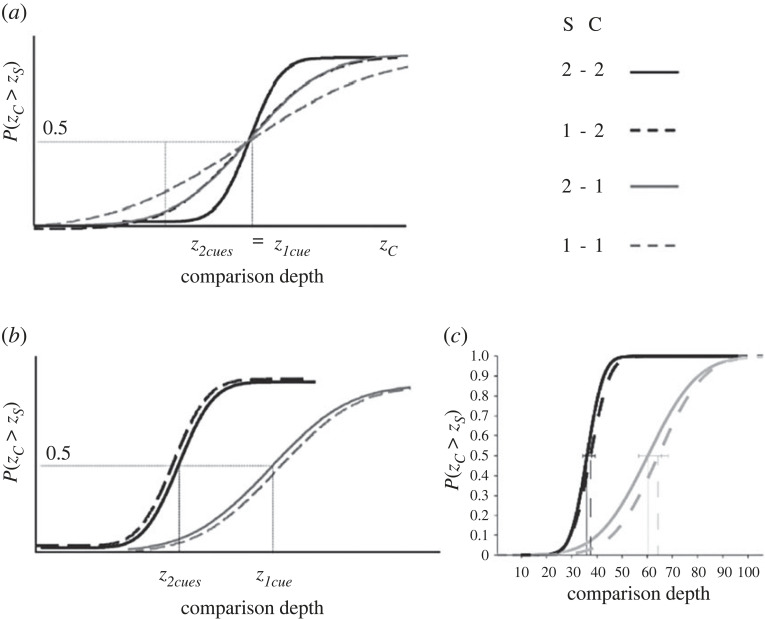
(*a*) MLE model predictions of the psychometric functions for a depth-discrimination task. In the legend, S indicates the standard stimulus and C the comparison stimulus. ‘2’ refers to the combined-cue stimuli and ‘1’ to the single-cue stimuli. The slope of the psychometric functions depends on both the standard and comparison stimuli. (*b*) Vector Sum model predictions. The slope of the psychometric functions depends only on the comparison stimuli. (*c*) Empirical data [70].

Using a similar experimental paradigm to the one illustrated above, I will extend the predictions of the MLE model to a discrimination task where single-cue or combined-cue stimuli are either assigned to be the fixed standard or the variable comparison. Figure 7*a* shows the psychometric curves predicted by the MLE model. First, since the MLE model assumes accurate outputs in all conditions, the PSE of the psychometric curves is predicted to be the same for all combinations of standard and comparison stimuli (figure 7*a*, PSEs: $z_{2\mathrm{cues}}=z_{1\mathrm{cue}}$). Second, as for the case of single-cue stimuli described in §3(b), the slope of the psychometric function depends on both the reliability of standard and comparison. We therefore expect: (i) the steepest slope when standard and comparison are both (the more reliable) double-cue stimuli (figure 7*a*, solid black); (ii) intermediate slopes when standard and comparison are different (figure 7*a*, dashed black and solid grey); and (iii) the shallowest slope when both standard and comparison are both (the less reliable) single-cue stimuli (figure 7*a*, dashed grey).

For the Vector Sum model, combining cues increases the depth-cue strength (figure 2*e*, equation (8)). Since the JND only depends on the cue strength, we expect steeper psychometric curves for combined-cue comparisons (figure 7*b*, black curves) and shallower psychometric curves for single-cue comparisons (figure 7*b*, grey curves). In both cases, the slope does not depend on the standard stimulus. We also expect that when the cue strengths of comparison and standard stimuli are different, the PSEs are also different, since depth estimates of combined-cue stimuli are predicted to be *larger* than those of single-cue stimuli because the strength of combined cues is larger (figure 2*e*, equations (6) and (7)). Therefore, the simulated depth $z_{2\mathrm{cues}}$ of combined stimuli must be smaller than the simulated depth $z_{1\mathrm{cue}}$ of single-cue stimuli to obtain a perceptual match (figure 7*b*). In a recent experiment, I tested these predictions with disparity and texture cues. The single-cue stimuli were random-dot stereograms simulating 3D bumps similar to those shown on figure 1. The combined-cue stimuli provided both texture and disparity information. In figure 7*c*, I show the results of a representative observer [70]. The results accurately match the predictions of the Vector Sum model and contradict the predictions of the MLE model.

These findings impose a radical re-interpretation of previous results on depth-discrimination experiments used as the strongest evidence in support of the MLE model. As mentioned before, previous results seem to indicate that the variance of combined-cue estimates is smaller than the variance of single-cue estimates as predicted by MLE (figure 1*c*, equations (4) and (5)) [15,30]. However, in these experiments, depth discrimination was studied separately for single-cue and combined-cue stimuli. Note, however, that in these experimental conditions the Vector Sum model makes identical predictions for the slope of the psychometric function (figure 7*a*,*b*) where the smaller JND observed for combined-cue discriminations is due to the larger combined-cue strength, which amplifies the response to distal depth changes (figure 2*e*, equations (7) and (8)). Most importantly, when strengths are expressed in terms of JNDs (figure 2*f*, equations (9) and (10)) the relationship between the predicted JND of the combined-cue stimulus and the JNDs of the single-cue stimuli is identical to the prediction of the MLE model (figure 2*c*, equation (5))^1^.

In summary, the Vector Sum model can predict previous results from depth-discrimination experiments used to confirm the predictions of the MLE model. However, the Vector Sum model can also predict the outcomes of untested experimental conditions where the cues displayed in the standard and comparison stimuli are different. In these newly tested conditions the predictions of the MLE model are falsified. From these results we can conclude that variability of depth judgements measured in depth-discrimination tasks does not reflect the output variability of 3D processing modules, as predicted by the MLE model. Instead, as predicted by the Vector Sum model, the JND is influenced by task-related noise and the cue strength.

## 4. Are 3D estimates from single-cues accurate? Evidence from a depth-matching experiment

The MLE model is based on the fundamental assumption that the output of depth modules is on average accurate (i.e. it is *unbiased*). The Vector Sum model, instead, assumes that individual outputs are only linearly related to distal 3D properties, but, in general, do not correspond the ground truth (i.e. they are *biased*). It is only the ‘lucky’ circumstance of encountering the ideal stimulus that yields a cue strength *k_i_* = 1 that will *coincidentally* produce a veridical output. Typically, cue strengths elicit under-estimation of 3D properties, but can in some specific viewing conditions also produce over-estimations (figure 2*d*, left panels, equations (D1)).

**Figure 8.**
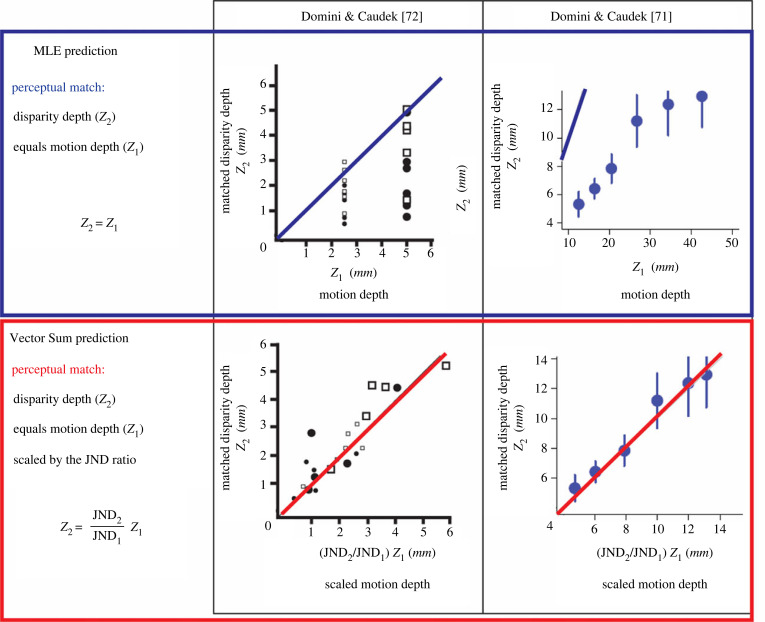
Results of two studies by Domini & Caudek ([71], right column, and [72], left column) where subjects matched a motion stimulus of depth *z*_1_ with a disparity stimulus. *z*_2_ is the disparity depth that yields a perceptual match. (Top) The MLE model predicts accuracy: a perceptual match is obtained when the disparity depth (*z*_2_) equals the motion depth (*z*_1_). This prediction, represented by a blue line, is shown on the two graphs where matched disparity depth is plotted as function of motion depth. The symbols on the left plot indicate individual observers. The symbols on the right plot represent data averaged across observers. The data show that responses are highly inaccurate and, therefore, in clear disagreement with the MLE predictions. (Bottom) The Vector Sum model predicts a perceptual match when the disparity depth (*z*_2_) is equal to the motion depth (*z*_1_) scaled by the JND ratio $(\;\mathrm{JN}D_{2}/\mathrm{JN}D_{1})$. This prediction, represented by the red lines, is shown on the two graphs where the same results shown on the top graphs have been replotted so that the matched disparity depth is shown as function of the scaled motion depth $(\;\mathrm{JN}D_{2}/{\mathrm{JND}}_{1})z_{1}$. The data clearly agree with the predictions of the Vector Sum model.

If the assumption of the MLE model is true then the perceived depth from one cue should match the perceived depth from a different cue as long as both cues specify the same 3D structure. Specifically, consider a depth-matching task where a fixed single-cue (e.g. motion) standard stimulus specifying a depth magnitude *z*_1_ is compared to a comparison stimulus specified by a different depth cue (e.g. disparity). The simulated depth of the comparison stimulus (*z*_2_) is varied until a depth match is obtained. If the single-cue outputs are unbiased, as assumed by the MLE model, then a perceptual match is obtained when $\;z_{1}=z_{2}$. By contrast, the Vector Sum model predicts that the distal depth values resulting in a perceptual match are not in general the same, but depend on the cue strength of each cue. Since perceived depth ${\hat{z}}_{1}=\;k_{1}z_{1}$ and ${\hat{z}}_{2}=\;k_{2}z_{2}$ (figure 2*d*, equation D1) a perceptual match is obtained when$\;k_{1}z_{1}=k_{2}z_{2}$, where *k*_1_ and *k*_2_ are the cue strengths. Therefore, since$\;z_{2}=(k_{1}/k_{2})z_{1}$, the depth match can be predicted from the cue strength ratio (*k*_1_/*k*_2_). Unfortunately, this ratio cannot be determined since the values of the cue strengths are unknown. Remember, however, that according to the Vector Sum model JNDs are inversely proportional to the cue strengths (figure 2f, equation (9)). Therefore, the ratio *k*_1_/*k*_2_ can be determined from the two discrimination thresholds JND_1_ and JND_2_ obtained in independent discrimination tasks performed on each cue separately. As a consequence, the ratio between the cue strengths is inversely proportional to the ratio between the JNDs: $k_{1}\;/k_{2}={\mathrm{JND}}_{2}/{\mathrm{JND}}_{1}$. Given this additional quantity, the prediction of the Vector Sum model is $z_{2}=({\mathrm{JND}}_{2}/{\mathrm{JND}}_{1})z_{1}$. I tested this prediction for motion and disparity cues in two separate studies [71,72] and indeed found that the perceptual match was not veridical as predicted by the MLE model. As can be seen in figure 8 (top), simulated depth from disparity (*z*_2_) producing a perceptual depth match is much smaller than the simulated depth from motion (*z*_1_). However, as can be seen in figure 8 (bottom), the simulated depth from motion scaled by the JND ratio (such that $z_{2}=({\mathrm{JND}}_{2}/{\mathrm{JND}}_{1})z_{1}$) accurately predicts the empirical data and therefore confirms the predictions of the Vector Sum model.

On a final note, these results not only reject the assumption of the MLE model that 3D perception is accurate, but also provide further converging evidence that JNDs do not measure the s.d. of single-cue outputs. In fact, according to the MLE theory, the PSEs estimated in a matching task and the JNDs resulting from discrimination tasks should be completely *unrelated parameters*. This is because the PSEs are estimates of the means of the likelihood functions associated with each cue (which should correspond to the ground truth) and the JNDs are estimates of the s.d. of these likelihood functions. By contrast, for the Vector Sum model, both PSEs and JNDs are univocally determined by the cue strength. The results shown in figure 8 clearly confirm this second interpretation of JNDs.

## 5. Predicting cue combination

The MLE and Vector Sum models make different predictions about the outcome of *cue integration* experiments. In reference to figure 1, consider an experiment exploring the combination of texture and shading information. In the single-cue condition, only one cue carries information about the 3D structure of distal objects. The bump in figure 1*b* viewed in a uniformly illuminated environment contains only a texture gradient. Conversely, if the material composition of the bump produces a negligible texture gradient but it is illuminated by a distant light source it carries only shading information (figure 1*c*). In the combined-cue conditions, both texture and shading are present (figure 1*a*).

The MLE model combines in a statistical optimal fashion the single-cue outputs through a weighted average, where the weights are inversely proportional to variance of the output noise (figure 2*b*, equations (1) and (2)). The perceived depth magnitude of a cue-combined stimulus is therefore predicted to be ‘in between’ the perceived depth magnitudes of the single-cue stimuli and closer to that of the more reliable cue. In the example of figure 1, the perceived depth of the texture-shading stimulus should be intermediate to the perceived depth of the shading-only and texture-only stimuli.

The Vector Sum model treats the single-cue outputs as orthogonal components of a multi-dimensional vector. The length of this vector is the combined-cue estimate (figure 2*e*, equations (6)–(8)). Therefore, the perceived depth magnitude of the combined-cue stimulus is always predicted to be *larger* than the perceived depth of single-cue stimuli. This phenomenon can be directly observed in figure 1 where the texture-shading stimulus appears deeper than the texture-only and shading-only stimuli.

**Figure 9.**
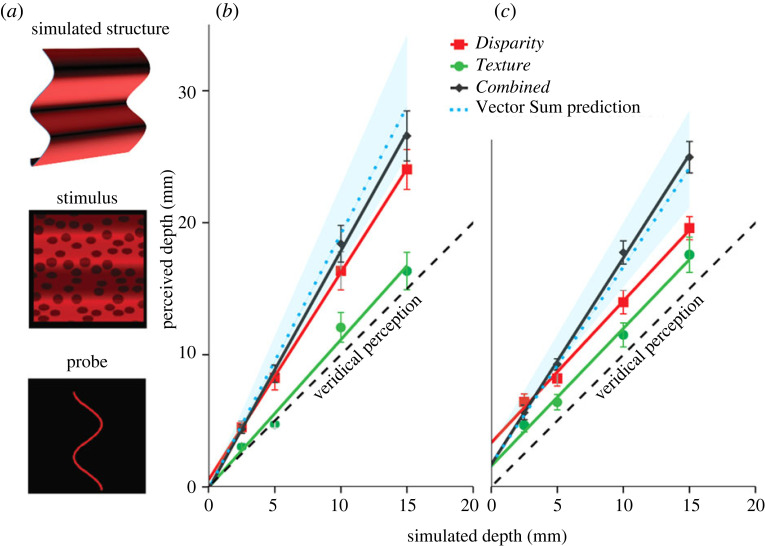
(*a*) The stimuli used in a study of cue integration simulated the projection of a sinusoidal corrugation in depth providing texture and disparity information [73]. The observers adjusted a two-dimensional sinusoidal probe until its amplitude matched the perceived amplitude of the sinusoidal depth corrugation. (*b*,*c*) Perceived depth as a function of simulated depth in the three experimental conditions: disparity (red), texture (green) and combined (black). The stimuli were seen at two distances: 40 cm (*b*) and 80 cm (*c*). The light-blue areas represent the 95% confidence intervals of the Vector Sum model predictions. The MLE model predicts that the combined-cue judgements should fall in between the single-cue judgements [73].

In a recent experiment where we studied the combination of texture and binocular-disparity information, we confirmed the predictions of the Vector Sum model [73]. Observers judged the depth of a sinusoidal corrugation by adjusting the amplitude of a two-dimensional sinusoidal probe in three conditions (figure 9*a*). In the *Texture* condition, only the texture cue was present. In the *Disparity* condition, we showed subjects a random-dot stereogram, so that only binocular disparities were present. In the *Combined* condition, both cues were present.

Figure 9 shows the results of the experiment repeated at two viewing distances (40 cm and 80 cm) [73]. Note first that at 40 cm the single-cue estimates differ from each other. Perceived depth from disparity (red) is larger than perceived depth from texture (green) and is significantly overestimated. However, depth in the combined condition (black) is even more overestimated. At 80 cm, perceived depth from disparity appears to be close to veridicality and close to perceived depth from texture, as seen by their matching slopes. But when the cues are combined, perceived depth is again larger and overestimated. These results are predicted by the Vector Sum model: the shaded light-blue areas on figure 9 indicate the confidence intervals of the predictions for the combined-cue results obtained directly from the single-cue data. The results clearly match this prediction and deviate systematically from a weighted average prediction of the MLE model (which should fall in-between the single-cue estimates) [73].

## 6. Concluding remarks

In this paper, I proposed a new computational theory of cue integration, termed IC, as an alternative to the current theories based on probabilistic inference. The IC theory is implemented with the Vector Sum model, a *deterministic* model that represents the outputs of independent processing modules as the components of a multi-dimensional vector. The norm of this vector is the estimate of a 3D property (e.g. depth, slant or curvature). This model maximizes the sensitivity to changes in distal properties while minimizing the undesired influence of nuisance scene parameters, such as the motion of the observer, illumination conditions, material composition of objects, etc. There are two main reasons why I argue that the Vector Sum model of depth-cue integration is a more viable alternative to the MLE models. First, the Vector Sum model is ***more parsimonious***, since it does not require two strong foundational assumptions of the MLE approach. The first assumption is that independent modules provide *accurate* estimates of 3D properties of distal objects. Instead, the Vector Sum model only postulates a *linear* mapping between distal properties and each module output. The second assumption of the MLE model is that the outputs of the single-cue modules are combined through a weighted average that favours the most reliable cues. This requires that each module also provides information about cue reliability. In order to hypothesize possible neural mechanisms that implement this combination process, it has been suggested that cortical neurons directly represent probability distributions and then combine those distributions in a statistical optimal fashion [19]. By contrast, the rule of combination of the Vector Sum model does not necessitate any estimate of the ‘quality’ of the output of single-cue modules.

Second, the IC model has ***more explanatory power***, since it predicts previous results in support of the MLE model and, most importantly, accounts for novel results that cannot be predicted by these models without the introduction of ad-hoc assumptions and priors [71–84]. From an empirical standpoint, the MLE model makes accurate predictions only in restricted experimental conditions, but fails to account for systematic biases in 3D judgements and does not explain simple phenomenological observations. The experimental tests that do agree with the MLE model hinge on the measurement and interpretation of discrimination thresholds, commonly termed JND. The methodological assumption of the MLE model is that JNDs are measures of the s.d. of the noise of 3D estimates. We showed that this methodological assumption, however, is incorrect, and provided new empirical evidence consistent with a radically different interpretation of JND. Through this new interpretation, the Vector Sum model can both predict previous data in support of the MLE model and new results incompatible with the MLE predictions.

It is important to highlight that both the IC theory and the probabilistic inference theories only specify how 3D estimates from separate modules are combined, but do not specify *how* the depth modules are implemented. This is certainly work for future research, but the way this problem should be approached must be guided by the assumptions the two theories make about the nature of the computation of depth modules and the output information. Take, for instance, the problem of deriving 3D shape from texture information. Probabilistic theories, and in particular Bayesian models, postulate that the visual system reverses the process of retinal projection to find among infinite structures in the world the most probable. This requires that the texture module embeds information about the statistical distribution of textures in the environment and also *a priori* probabilities of 3D structures in the world. If this information is correct then the module can compute the truthful probability distribution of 3D shapes that have generated a given visual input. In reference again to figure 1, this means that this output would generate a sharp probability distribution for the texture in figure 1*b* and a wide distribution for the texture in figure 1*d*. Moreover, the peaks of these distributions are expected to vary across views, with small variations for the reliable texture (figure 1*b*) and large variations for the unreliable texture (figure 1*d*). However, both outputs are expected to be on average veridical. Although attempts have been made in the past to develop probabilistic models of shape from texture, these have been confined to the very special case of planar surfaces [4,23]. In general, there are no existing working models of shape from texture that predict human behaviour. In contrast with probabilistic theories, the IC theory postulates the existence of depth modules that output a 3D estimate that is only linearly related to the ground truth, but it is not in general accurate. Unlike the probabilistic models, this estimate is stable across multiple views of the same type of stimulus. What it is expected, instead, is that the magnitude of this response depends on the strength of the cue. In the example of figure 1, the texture module will output a large response for the texture in figure 1*b* and a weak response for the texture in figure 1*d*. In contrast with the probabilistic models, the output estimate of depth modules does not carry any information about the ‘quality’ of the visual input, such that the response to a weak cue (the texture cue in figure 1*d*) and the response to a strong cue projected from a shallow surface are indistinguishable. Therefore, according to the IC theory, single-cue modules should be attuned to image properties that are *invariant* with the nuisance scene parameters (e.g. the material property that determines the surface texture). A consequence of this assumption is that, in stark contrast with the MLE model, the visual system does not ‘weigh’ the outputs of individual modules based on some measure of ‘reliability’ or strength of cues, since such information is absent from the module output. In other words, the visual system is *blind* to the distal causes of the visual input and simply combines all the module outputs through a vector sum. It is the logic of the vector sum regime that guarantees that the combined output will always optimally track the underlying distal depth variable, regardless of the strength of the individual cues.

In a final note, it should be emphasized that although the two theories do not specify how depth modules may be learnt, the depth modules postulated by the IC theory do not necessarily require information about the ground truth for their mechanisms to be learnt. Instead, *invariant* properties of independent image signals that covary with distal properties must also necessarily covary among themselves and, likewise, covary with signals arising from other modalities such as touch or proprioception. Discovering these covariations may be a plausible learning process for a biological system that never has direct access to the distal geometric structure of the environment [85,86].

## Data Availability

This article has no additional data.

## Endnote

1 This can be verified by combining equations (9) and (10) of figure 2*f.*

## Appendix A

Appendix 1: *The Vector Sum model maximizes the Signal-to-Noise-Ratio*. For simplicity consider only two signals $s_{1}=\lambda_{1}z$ and $s_{2}=\lambda_{2}z$, where $\lambda_{i}$ are unknown multipliers depending on confounding variables and *z* is the magnitude of the 3D property. These signals are the visual system encoding of 3D information from single cues. We seek an estimate ${\hat{z}}_{C}=f(s_{1},s_{2})$ (1) proportional to *z* and (2) most sensitive to 3D information and least sensitive to random fluctuations $\varepsilon_{i}$ of $\lambda_{i}$. If $\lambda_{i0}$ is the unperturbed value of $\lambda_{i}$ then $\lambda_{i}=\lambda_{i0}+\;\varepsilon_{i}$ and $s_{i0}=\lambda_{i0}z$. We assume small random perturbations due to changes in viewing conditions such that $\varepsilon_{i}$ are Gaussian distributions with zero mean and standard deviations $\sigma_{i}.\;$Taking the derivative of

$$\frac{df(s_{1},s_{2})\;}{dz}=\frac{df\;\;}{ds_{1}}(\lambda_{10}+\;\varepsilon_{1})+\frac{df\;\;}{ds_{2}}(\lambda_{20}+\;\varepsilon_{2}),$$

where d*f*/d*s_i_* are calculated at $s_{i0}$, we observe a signal term $S=f_{1}\lambda_{10}+f_{2}\lambda_{20}$ (where $f_{i}=df\;\;/ds_{i}$) and a noise term $E=f_{1}\varepsilon_{1}+f_{2}\varepsilon_{2}$ having s.d. $\sigma_{E}=\sqrt{\;f_{1}^{2}\sigma_{1}^{2}+f_{2}^{2}\sigma_{2}^{2}}$. If we minimize the Noise to Signal Ratio $NSR=\frac{\sigma_{E}\;}{S}$ with respect to *f_i_* (by solving for *f_i_* the equation $\mathrm{dSNR}\;/df_{i}=0$), we find that the first derivatives of the function are $df\;/ds_{i}\propto \lambda_{i0}\;/\sigma_{i}^{2}$. It can be shown that the derivatives $d{\hat{z}}_{C}\;/ds_{i}\;$ of the equation ${\hat{z}}_{C}=\beta \sqrt{{\left(\frac{s_{1}\;}{\sigma_{1}}\right)}^{2}+{\left(\frac{s_{2}\;}{\sigma_{2}}\right)}^{2}}$ (calculated at $\;s_{i0}$) meet this requirement. By substituting $k_{i}=\beta (\lambda_{i}/\sigma_{i})$, we obtain the Vector Sum equation$\;{\hat{z}}_{C}=\sqrt{{\left(k_{1}z\right)}^{2}+{\left(k_{2}z\right)}^{2}}$ (easily generalizable to *n* signals).
